# Investigating the reciprocity between cognition and behavior in adaptation to large-scale disasters

**DOI:** 10.1038/s44184-023-00037-8

**Published:** 2023-12-04

**Authors:** Tiffany Junchen Tao, Tsz Wai Li, Li Liang, Huinan Liu, Wai Kai Hou

**Affiliations:** 1 Centre for Psychosocial Health, The Education University of Hong Kong, Hong Kong SAR, China; 2 Department of Psychology, The University of Hong Kong, Hong Kong SAR, China; 3 Department of Special Education and Counselling, The Education University of Hong Kong, Hong Kong SAR, China; 4 Department of Psychology, The Education University of Hong Kong, Hong Kong SAR, China

**Keywords:** Psychiatric disorders, Risk factors, Human behaviour, Epidemiology

## Abstract

Cognition and behavior could reciprocally impact each other and together determine mental health amid large-scale disasters such as COVID-19. This study reports a six-month cohort study of a population-representative sample of Hong Kong residents (*N* = 906) from March–August 2021 (T1) to September 2021–February 2022 (T2). Cross-lagged panel analyses reveal that T1 poor behavioral functioning as indicated by high daily routine disruptions is inversely associated with T2 cognitive adaptation as indicated by self-efficacy and meaning-making but not vice versa. T1 routine disruptions but not cognitive adaptation are positively associated with T2 probable depression/anxiety. The positive link between T1 routine disruptions and T2 probable disorders is mediated by poor cognitive adaptation at T2. The present findings suggest that upholding daily behavioral functioning relative to positive states of mind could have a more pivotal role in mental health amid large-scale disasters. Future studies can test interventions that enhance the sustainment of regular daily routines.

## Introduction

Cognitive and behavioral aspects of adaptation are considered targets of modification and essential for building strengths and determining mental health^1^ across different levels of social, behavioral, cognitive, and neurobiological functioning for adaptive adjustment^2^. These factors do not exhibit their effects in isolation, because changes in some core factors will positively influence other related factors^3^ and together improve adaptation outcomes. There is a need to investigate and compare protective cognitive and behavioral factors for population mental health assessment and intervention under large-scale disasters.

The Conservation of Resources (COR) theory suggests that resource change dictates mental health in stress adaptation^4^. Ongoing demands are offset by mobilizing at least three types of resources, namely personal (e.g., personalities and self-concepts that are integral to oneself), social (e.g., social support and relationship quality), and material (e.g., money or tangible belongings that could be utilized) resources.

Cognitive adaptation is the attempt to engage in cognitively adaptive efforts that enable individuals to resume and go beyond pre-existing levels of functioning in response to setbacks and tragedies^5,6^. Both theoretical and empirical literature suggested that perceived self-efficacy and life meaning as examples of cognitive adaptation predict adaptive behavioral adjustment and better mental health. Self-efficacy denotes confidence in one’s capability to cope with stressors^7^ and relates to increased levels of physical activity in adaptation to chronic stressors such as medical conditions^8^ and low socioeconomic status^9^. Among the general population, individuals with higher self-efficacy were found to report better sleep hygiene and fewer sleep problems^10^ and perform more oral health care behaviors^11^. Meaning in life denotes a sense of feeling that one’s life is significant, purposeful, and coherent^12^. Awareness of meaning in life has been suggested to activate self-regulatory processes that enhance physical and psychological health^13^. People with cardiovascular disease who had higher levels of meaning in life also reported higher levels of health awareness and positive lifestyle such as smoking cessation and body weight management^14^.

There is a concurrent body of evidence suggesting the adaptive priority of behavioral processes. The social zeitgeber model proposed that daily activities (e.g., social contact, meal/bedtime, work/studies, leisure) are regularized to form social rhythms that maintain physical and mental health under normal circumstances and reduce the risk of severe mental health problems such as bipolar disorder^15^. The Drive to Thrive (DTT) theory similarly argued that sustainment of regular daily routines is one of the core processes that contribute to psychological resilience (i.e., non-significant psychological distress or psychiatric symptoms over time) across trauma and chronic stress conditions^16^.

Data from experience sampling methodology further revealed that more regular daily routines were associated with higher levels of meaning in life, suggesting that everyday activities provide contexts and pathways towards desired goals and a sense of fulfillment in the long run^12^. Regularities in basic daily activities have also been found to predict higher levels of sleep efficacy, which in turn were associated with lower depressive and anxiety symptoms^17^. Engagement in leisure activities was inversely associated with depressive symptoms through increased perceptions of predictability, controllability, and meaningfulness^18^. Regularity per se could be more strongly associated with cognitive and emotional adaptation, for sustainment of regular routines could build an overall well-structured daily life that effectively buffers individuals from the negative mental health impact of stressful events^16^.

Beyond determining the adaptive priority between cognitive and behavioral processes leading to psychological resilience, theoretical and empirical evidence also points to possible reciprocal facilitation between the two processes. Mental health outcomes can be seen as a product of orchestrated effects of various resilience factors^3^. These factors exist within an interrelated system where they form positive mutual connections and enhancements critical to adaptive psychological adjustment. The process of resilience is therefore also the process of accumulation and optimization of resilience factors^2^.

In a similar vein, persons with more resources tend to experience resource gain, while persons with fewer resources are more likely to suffer from resource loss^4^. Resource caravan passageways refer to contexts that enrich, foster, and conserve coping resources, or reduce, deter, and deplete resources, depending on whether individuals are able to maintain or create the contexts that are conducive to resource gain^19^. Resource multiplication theory also suggested that the positive association between socioeconomic resources such as education and mental health is stronger for individuals with more resources^20^. Individuals who are more advantaged, relative to those who are not, are more likely to experience a larger benefit of coping resources on their mental health. Recovery and sustainability have further been suggested to be two parallel aspects of human stress resilience^21^. Recovery refers to the capacity to recover from negative psychophysiological sequelae brought about by stressful events, whereas sustainability refers to the capacity to maintain or gain positive physical and psychological health over the course of adaptation. The recovery and sustainability pathways interact with each other while developing independently in a stress process^22^. To our knowledge, most existing cross-sectional and prospective studies on cognitive and/or behavioral processes in stress adaptation were conducted with the assumption of a unidirectional – not bidirectional – sequential pathway between the two processes. A more holistic approach to investigating resilience factors is warranted and will likely generate important insights.

A meta-analysis was conducted on 66 studies (221,970 respondents) and concluded that the pandemic was responsible for a pooled prevalence of 30–40% for common mental health problems such as depression, anxiety, insomnia, and general distress in the acute phase across different populations^23^. Cognitive adaptation has been associated with lower levels of depressive and anxiety symptoms in COVID-19^24^. Such personal resources are particularly important to mental health under COVID-19, when social resources are limited by infection control rules such as lockdown and quarantine^25^. Amid COVID-19, both self-efficacy and meaning in life have been positively related to engagement in health-protective behaviors^26,27^, suggesting that resources on different levels could collectively influence behavioral outcomes. During lockdown, structured daily routines such as regularized physical activities buffered individuals of depressive, anxiety, and stress symptoms and negative affect^28^. On the flip side, loss of the original work, exercise, and social schedules was found to compromise coping abilities, which in turn was associated with more emotional symptoms and poorer psychological well-being^29^.

This study aims to investigate the interrelations between cognitive and behavioral processes and their associations with mental health outcomes in a prospective population-representative cohort in Hong Kong during COVID-19. We hypothesized that cognitive adaptation and daily routine disruptions will be inversely associated with each other both concurrently and prospectively. Cognitive adaptation will be negatively associated whereas routine disruptions will be positively associated with probable psychiatric conditions. We also tested the mediating effects of cognitive adaptation and routine disruptions in the associations of each other with the outcomes.

## Methods

### Respondents and procedure

This prospective study collected population-representative data at two time points, with a follow-up duration of six months for all respondents (T1 = March–August 2021, T2 = September 2021–February 2022). The methods were performed in accordance with relevant guidelines and regulations and approved by the Ethics Review Committee of The Education University of Hong Kong (2019–2020–0224 and 2020–2021–0277). Telephone surveys were conducted among Hong Kong Chinese aged ≥15 years by interviewers who had received prior formal training. Oral informed consent was obtained from each participant prior to the survey. The sampling procedure of the current study closely followed that of other large-scale local prospective cohort studies. The final sample included 906 individuals. The cooperation (eligible individuals invited) and response (invited individuals complying with acceptable standards) rates at T2 (98.48% and 68.64%) were good^30^ (Supplementary Notes1–2). All analyses were weighted to account for non-response. All respondents received supermarket coupons with a face value HK$100 (≈US$13) as compensation for participation.

### Measures

#### Cognitive adaptation

Cognitive adaptation consisted of self-efficacy and meaning-making for their relations to adaptive behavioral adjustment and better mental health under large-scale disasters. Self-efficacy was measured using the Chinese version of the 6-item General Self-Efficacy Scale (GSE-6)^31^. Respondents rated their perceived ability to take control under stressful conditions *over the past month* on a 4-point Likert scale (1 = strongly disagree, 4 = strongly agree). Meaning-making was measured with the Positive Reinterpretation and Growth subscale on the COPE^32^ and the Emotional Processing Scale^33^ based on previous studies^34^. The Positive Reinterpretation and Growth subscale consisted of four items regarding one’s tendency to approach situations in a positive way, while the Emotional Processing Scale consisted of four items concerning one’s attempts to understand their own emotional reactions to situations. Respondents rated each item on a 4-point Likert scale (1 = not at all, 4 = always) with reference to their experience *in* *the past month*. We conducted confirmatory factor analysis (CFA) to evaluate the structural validity of this single composite measure (Supplementary Note 3). Cronbach’s αs were 0.888 and 0.871 at T1 and T2, respectively, suggesting good internal consistency of the items.

#### Disruptions to primary and secondary routines

Daily routine disruptions were measured with eight items on the Sustainability of Living Inventory (SOLI)^35,36^, four measuring primary routines (i.e., hygiene, eating, sleep, duties at home) and four measuring secondary routines (i.e., leisure at home, exercising, social activities, work/study involvement). Respondents indicated how much the regularity of each behavior was disrupted *over the past two weeks* on an 11-point scale (0 = no disruptions, 10 = high level of disruptions). Mean scores were calculated for primary and secondary routines, and then summed to generate a total score, with higher scores indicating higher levels of disruptions. CFA was conducted on the routine items to test the model fit of the single-factor model (Supplemental Note 3). Internal consistency of the items was good over time (T1: α = 0.804; T2: α = 0.811).

#### Probable depression

Depressive symptoms were assessed using the 9-item Patient Health Questionnaire (PHQ-9)^37^. Respondents indicated on a 4-point scale (0 = not at all, 1 = on several days, 2 = on more than half of the days, 3 = nearly every day) the frequency of experiencing depressive symptoms *over the past two weeks*. Higher total scores indicated greater severity of depressive symptoms (range = 0–27). PHQ-9 showed high internal consistency at T1 (α = 0.885) and T2 (α = 0.880) administrations. A cut-off score of 10 was adopted to indicate probable depression and recode the scores into a dichotomized scale (1 = yes, 0 = no)^38^.

#### Probable anxiety

Anxiety symptoms were assessed using the 7-item Generalized Anxiety Disorder scale (GAD-7)^39^. Respondents indicated the frequency they experienced anxiety symptoms *over the past two weeks* on a 4-point scale (0 = not at all, 1 = on several days, 2 = on more than half of the days, 3 = nearly every day). A total score was calculated, with higher scores indicating greater severity of anxiety symptoms (range = 0–21). The GAD-7 showed high internal consistency at T1 (α = 0.934) and T2 (α = 0.940) administrations. A cut-off score of 10 was used to indicate probable anxiety and recode the scores into a dichotomized scale (1 = yes, 0 = no)^40^.

#### Demographics

At T1, gender, age, marital status, education level, employment status, and monthly household income were recorded.

### Statistical analysis

All missing data (<1%) were handled with multiple imputation. For cognitive adaptation, scores on self-efficacy and meaning-making were standardized and averaged to indicate cognitive adaptation, whereas for routine disruptions, scores on disrupted primary and secondary routines were summed and standardized to indicate overall daily routine disruptions^22^. Cognitive adaptation and routine disruptions were then recoded into two groups (1 = high, 0 = low) following median split. At both T1 and T2, correlations between cognitive adaptation and daily routine disruptions were comparable with or lower than those between self-efficacy and meaning-making and between primary and secondary routine disruptions, suggesting empirical divergence between cognitive adaptation and overall routine disruptions.

Two cross-lagged panel models were constructed for probable depression and probable anxiety within M*plus* (Version 8.3). In each model, we tested the concurrent and prospective associations between cognitive adaptation, daily routine disruptions, and probable psychiatric conditions at T1 and T2, controlling for sociodemographic covariates. Directional relationships were inferred statistically in the presence of cross-lagged effects, that is, when the coefficient for the parameter linking a variable *X*_*1*_ at T1 with another variable *Y*_*2*_ at T2 was significantly different from zero. The temporal ordering of the three variables was inferred in the presence of simultaneous consideration of potential reciprocal cross-lagged effects (i.e., through comparing the coefficients for parameters for both *X*_*1*_ to *Y*_*2*_ and *Y*_*1*_ to *X*_*2*_) as well as the stability of each variable itself over time within autoregressive pathways (i.e., from *X*_*1*_ to *X*_*2*_).

To establish mediating effects, two additional sets of path analyses were conducted, testing whether T2 routine disruptions (or cognitive adaptation) mediated the prospective associations between T1 cognitive adaptation (or routine disruptions) and T2 probable psychiatric conditions. T1 probable psychiatric condition and sociodemographic covariates were adjusted for. In both cross-lagged panel and path analyses, the weighted least square mean and variance adjusted (WLSMV) estimator were used, whereas model fit was assessed by a combination of residual mean squared error of approximation (RMSEA) (<0.08), standardized root mean square residual (SRMR) (<0.08), comparative fit index (CFI) (>0.90), and Tucker-Lewis index (TLI) (>0.90)^41^. Sensitivity analyses were conducted adopting continuous variables for all main analyses.

### Reporting summary

Further information on research design is available in the Nature Research Reporting Summary linked to this article.

## Results

### Respondents and Prevalence

Demographics of the respondents (*N* = 906) are summarized in Table 1. The prevalences of probable depression (i.e., PHQ-9 ≥ 10) and anxiety (i.e., GAD-7 ≥ 10) were 21.41% and 17.11% at T1, and 24.94% and 18.98% at T2.

**Table 1:** Descriptive characteristics of the current sample.

Variable	*n*	%
*Gender*		
Male	468	51.66
Female	438	48.34
*Age*		
65 or above	112	12.36
55–64	102	11.26
45–54	118	13.02
35–44	183	20.20
25–34	226	24.94
15–24	165	18.21
*Marital status*		
Married	433	47.79
Single/divorced/widowed	473	52.21
*Education*		
Tertiary or above	586	64.68
Secondary	284	31.35
Primary or below	36	3.97
*Employment*		
Employed	630	69.54
Unemployed/dependent	276	30.46
*Monthly household income*		
80,000 or above	116	12.80
60,000–79,999	102	11.26
40,000–59,999	187	20.64
20,000–39,999	247	27.26
19,999 or below	254	28.04
*T1 Cognitive adaptation*		
Low	432	47.68
High	474	52.32
*T2 Cognitive adaptation*		
Low	430	47.46
High	476	52.54
*T1 Routine disruptions*		
Low	453	50.00
High	453	50.00
*T2 Routine disruptions*		
Low	437	48.23
High	469	51.77
*T1 Probable depression*		
No	712	78.59
Yes	194	21.41
*T2 Probable depression*		
No	680	75.06
Yes	226	24.94
*T1 Probable anxiety*		
No	751	82.89
Yes	155	17.11
*T2 Probable anxiety*		
No	734	81.02
Yes	172	18.98

US$1 ≈ HK$7.80. Cognitive adaptation is a composite index constituting self-efficacy and meaning-making; Low *vs*. high groups were categorized based on median split. Routine disruptions is a composite index constituting disruptions to primary and secondary routines; Low *vs*. high groups were categorized based on median split. Probable depression was defined by scores of 10 or above on the 9-item Patient Health Questionnaire (PHQ-9). Probable anxiety was defined by scores of 10 or above on the 7-item Generalized Anxiety Disorder scale (GAD-7).

### Cross-lagged panel analyses

**Fig. 1:**
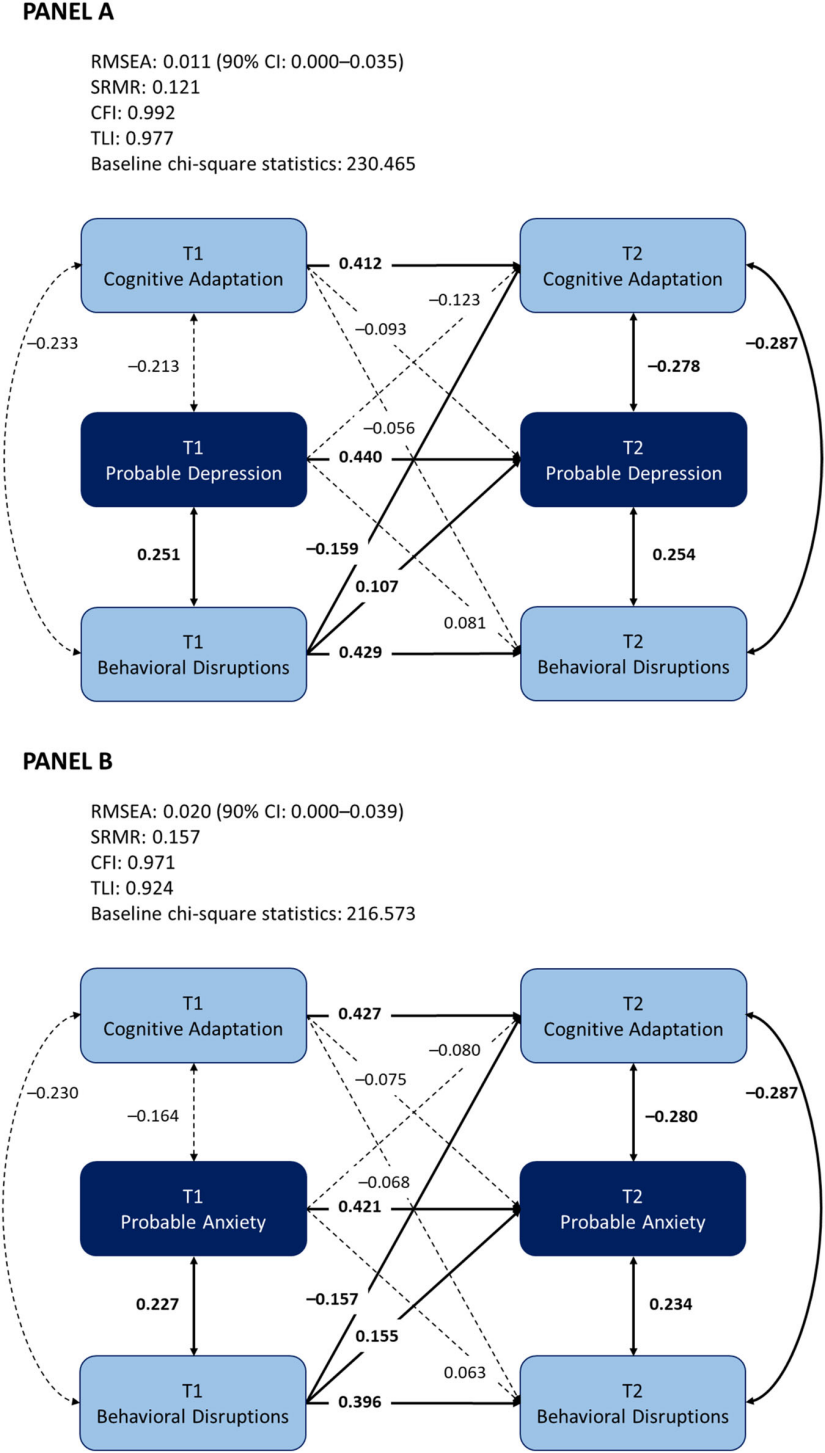
Cross-lagged panel model for cognitive adaptation, routine disruptions, and psychopathology. **A** Probable depression. **B** Probable anxiety. Cognitive adaptation included self-efficacy and meaning-making; routine disruptions included primary and secondary routine disruptions. Demographics covariates were adjusted for in the cross-lagged panel model. The full figure including demographics covariates is available from the corresponding author.

Figure 1 presents the cross-lagged models of cognitive adaptation, routine disruptions, and probable depression and anxiety.

The model for probable depression demonstrated good data-model fit (RMSEA = 0.011, 90% CI [0.000, 0.035], SRMR = 0.121, CFI = 0.992, TLI = 0.977). All autoregressive paths were statistically significant (*p*s ≤ 0.024). The cross-lagged paths were significant between T1 routine disruptions and T2 cognitive adaptation (*β* = –0.159, 95% CI [–0.312, –0.006], *p* = 0.042) and between T1 routine disruptions and T2 probable depression (*β* = 0.107, 95% CI [0.010, 0.204], *p* = 0.030). The cross-lagged paths were not significant between T1 cognitive adaptation and T2 routine disruptions (*β* = –0.056, 95% CI [–0.152, 0.040], *p* = 0.256) or T2 probable depression (*β* = –0.093, 95% CI [–0.213, 0.027], *p* = 0.128), or between T1 probable depression and T2 cognitive adaptation (*β* = –0.123, 95% CI [–0.272, 0.025], *p* = 0.103) or routine disruptions (*β* = 0.081, 95% CI [–0.014, 0.176], *p* = 0.093). Detailed statistical results are summarized in Table 2. Results of the sensitivity analysis based on continuous variables are presented in Supplementary Table 1.

**Table 2:** Cross-lagged analyses examining the autoregressive and cross-lagged effects of cognitive adaptation, routine disruptions, and probable depression.

Effect	Variables	*β* [95% CI]
Autoregressive	T1 Cognitive adaptation → T2 Cognitive adaptation	0.412 [0.054, 0.771]*
	T1 Routine disruptions → T2 Routine disruptions	0.429 [0.254, 0.604]***
	T1 Probable depression → T2 Probable depression	0.440 [0.352, 0.528]***
Cross-lagged	T1 Cognitive adaptation → T2 Routine disruptions	–0.056 [–0.152, 0.040]
	T1 Routine disruptions → T2 Cognitive adaptation	–0.159 [–0.312, –0.006]*
	T1 Cognitive adaptation → T2 Probable depression	–0.093 [–0.213, 0.027]
	T1 Routine disruptions → T2 Probable depression	0.107 [0.010, 0.204]*
	T1 Probable depression → T2 Cognitive adaptation	–0.123 [–0.272, 0.025]
	T1 Probable depression → T2 Routine disruptions	0.081 [–0.014, 0.176]

*p < 0.050, *** *p* < 0.001.

Probable depression was defined by scores of 10 or above on the 9-item Patient Health Questionnaire (PHQ-9); Cognitive adaptation and routine disruptions were binary variables (based on median split). Results of the sensitivity analysis based on continuous variables are presented in Supplementary Table 1.

Similarly, the model for probable anxiety demonstrated good data-model fit (RMSEA = 0.020, 90% CI [0.000, 0.039], SRMR = 0.157, CFI = 0.971, TLI = 0.924). All autoregressive paths were statistically significant (*p*s ≤ 0.011). The cross-lagged paths were significant between T1 routine disruptions and T2 cognitive adaptation (*β* = –0.157, 95% CI [–0.296, –0.018], *p* = 0.027), and between T1 routine disruptions and T2 probable anxiety (*β* = 0.155, 95% CI [0.058, 0.252], *p* = 0.002). The cross-lagged paths were not significant between T1 cognitive adaptation and T2 routine disruptions (*β* = –0.068, 95% CI [–0.165, 0.030], *p* = 0.173) or T2 probable anxiety (*β* = –0.075, 95% CI [–0.176, 0.026], *p* = 0.146), or between T1 probable anxiety and T2 cognitive adaptation (*β* = –0.080, 95% CI [–0.196, 0.037] *p* = 0.180) or routine disruptions (*β* = 0.063, 95% CI [–0.029, 0.154], *p* = 0.180). The detailed statistics are summarized in Table 3. Results of the sensitivity analysis based on continuous variables are presented in Supplementary Table 2.

**Table 3:** Cross-lagged analyses examining the autoregressive and cross-lagged effects of cognitive adaptation, routine disruptions, and probable anxiety.

Effect	Variables	*β* [95% CI]
Autoregressive	T1 Cognitive adaptation → T2 Cognitive adaptation	0.427 [0.096, 0.758]*
	T1 Routine disruptions → T2 Routine disruptions	0.396 [0.258, 0.534]***
	T1 Probable anxiety → T2 Probable anxiety	0.421 [0.321, 0.520]***
Cross-lagged	T1 Cognitive adaptation → T2 Routine disruptions	–0.068 [–0.165, 0.030]
	T1 Routine disruptions → T2 Cognitive adaptation	–0.157 [–0.296, –0.018]*
	T1 Cognitive adaptation → T2 Probable anxiety	–0.075 [–0.176, 0.026]
	T1 Routine disruptions → T2 Probable anxiety	0.155 [0.058, 0.252]**
	T1 Probable anxiety → T2 Cognitive adaptation	–0.080 [–0.196, 0.037]
	T1 Probable anxiety → T2 Routine disruptions	0.063 [–0.029, 0.154]

Probable anxiety was defined by scores of 10 or above on the 7-item Generalized Anxiety Disorder scale (GAD-7); Cognitive adaptation and routine disruptions were binary variables (based on median split). Results of the sensitivity analysis based on continuous variables are presented in Supplementary Table 2.

*p < 0.050, **p < 0.010, ***p < 0.001.

### Path analyses

Path models were constructed to test the mediating effect of T2 daily routine disruptions on the associations between T1 cognitive adaptation and T2 probable psychiatric conditions and the mediating effect of T2 cognitive adaptation on the associations between T1 daily routine disruptions and T2 probable psychiatric conditions (Supplementary Fig. 1). Both the models for probable depression (RMSEA = 0.044, 90% CI [0.020, 0.070], SRMR = 0.040, CFI = 0.977, TLI = 0.918) and probable anxiety (RMSEA = 0.040, 90% CI [0.005, 0.072], SRMR = 0.026, CFI = 0.988, TLI = 0.946) demonstrated good data-model fit. The effect of T1 routine disruptions on T2 probable depression/anxiety was fully mediated by T2 cognitive adaptation (*β* = 0.032, 95% CI [0.006, 0.058], *p* = 0.017; *β* = 0.036, 95% CI [0.006, 0.066], *p* = 0.018). Meanwhile, T1 cognitive adaptation demonstrated neither a direct nor an indirect effect on T2 probable depression/anxiety. The results of the direct and indirect effects are summarized in Table 4. Results of the sensitivity analysis based on continuous variables are presented in Supplementary Table 3.

**Table 4:** Path analyses examining the mutual mediating mechanisms of cognitive adaptation and routine disruptions on probable depression/anxiety.

Outcome	Direct/Indirect effects	Direct/Indirect effect *β* [95% CI]
T2 Probable depression	T1 Cognitive adaptation	0.021 [–0.078, 0.120]
	T1 Cognitive adaptation → T2 Routine disruptions	–0.009 [–0.025, 0.007]
	T1 Routine disruptions	–0.042 [–0.149, 0.065]
	T1 Routine disruptions → T2 Cognitive adaptation	0.032 [0.006, 0.058]*
T2 Probable anxiety	T1 Cognitive adaptation	0.060 [–0.049, 0.170]
	T1 Cognitive adaptation → T2 Routine disruptions	–0.011 [–0.027, 0.006]
	T1 Routine disruptions	0.024 [–0.097, 0.145]
	T1 Routine disruptions → T2 Cognitive adaptation	0.036 [0.006, 0.066]*

Probable depression was defined by scores of 10 or above on the 9-item Patient Health Questionnaire (PHQ-9); Probable anxiety was defined by scores of 10 or above on the 7-item Generalized Anxiety Disorder scale (GAD-7); Cognitive adaptation and routine disruptions were binary variables (based on median split). Results of the sensitivity analysis based on continuous variables are presented in Supplementary Table 3.

*p < 0.050.

## Discussion

This two-wave cross-lagged panel analysis aims to investigate the reciprocal associations between cognitive and behavioral adjustment and the mediating effect of each on the other’s associations with prospective probable psychiatric conditions amid the COVID-19 pandemic. Our findings were largely comparable between probable depression and probable anxiety. Partially consistent with our expectations, only daily routine disruptions (at T1) displayed cross-lagged effects on both cognitive adaptation and probable depression/anxiety (at T2), meaning that daily routine disruptions could predict lower cognitive adaptation and higher odds of probable depression/anxiety but not the other way round. Further path analyses only identified a significant mediating effect of T2 cognitive adaptation on the association between T1 daily routine disruptions and T2 probable depression/anxiety. The COVID-19 pandemic disrupted perceptions of the self and the world as well as obstructed daily lives. The current study demonstrated elements that could be rebuilt to aid adaptive adjustment.

Our findings suggested a fundamental role of behavioral processes preceding emotional and cognitive adjustments in face of chronic stressors. Under COVID-19 when the maintenance of regular activities was seriously challenged, daily routine disruptions were positively associated with depressive or anxiety symptoms^29^, whereas successful maintenance of daily routines served as one of the protective factors against mental ill health^28^. Theoretically, the current results provided further prospective empirical evidence supporting the conceptual and clinical utility of the social zeitgeber model and the Drive to Thrive (DTT) theory^15,16^, with both proposing that regularity in daily routines could be seen as a necessary condition for emotional adjustment under chronic stress and trauma.

Conversely, we did not find significant prospective positive associations of probable depression/anxiety with subsequent daily routine disruptions. Our findings showed that daily routine disruptions are conceptually different from behavioral symptoms (e.g., changes in eating, sleep, or physical activity) that have long been considered as consequential to mood disorders^42^. Daily routine disruptions are the antecedent, not secondary behavioral manifestation, of probable depression/anxiety.

Based on the current significant association between T1 daily routine disruptions and T2 cognitive adaptation, behavioral processes could be considered as a fundamental process that precedes cognitive adaptation. There is a growing body of prospective evidence suggesting a close positive link of regularity in daily routines with self-efficacy^43^ or meaning in life^12^. Performance of routine behaviors have been found to relate to more feelings of comfort, safety, and confidence across real-life and laboratory settings^44^. For families with children suffering physical^45^ or mental^46^ health issues, routines were perceived as the bedrock on which family members supported one another, and children perceived themselves as able to accomplish tasks and achieve normal functioning levels, despite their health challenges. Daily routines function not only as a mere collection of activity schedules but also as an externalized form of coping under life difficulties.

The Conservation of Resources (COR) theory suggests that resource gain begets further resource gain, putting an individual within a positive cycle of resource accumulation^4^. Considering the unidirectional cross-lagged effect of daily routine disruptions on cognitive adaptation in conjunction with their significant cross-sectional associations among different factors, our results serve as a concrete illustration of how various resilience factors dynamically interact with one another through orchestrated effects to form a knitted network of resources^2,3^. Different types of daily routines and disruptions could demonstrate differential associations with cognitive adaptation, which could both be targets of intervention for positive adaptation^3^.

In the family routines literature, for example, it is suggested that household routines likely provide a context for children to experience belonging, acquire values/norms, improve self-regulation, and perceive predictability^47^. Our current findings similarly suggested that regularity of daily routines could form a behavioral context conducive to resilience outcomes through their positive associations with cognitive adaptation^16,35^. Broadly speaking, our results resonated theoretical perspectives on the priority to establish a resourceful environment over the pure cultivation of internal characteristics^48^, because securing the right context could foster the development of positive inner qualities, forming, in turn, a positive loop for psychological resilience.

On the other hand, we did not identify a significant association between T1 cognitive adaptation (i.e., combined self-efficacy and meaning-making) and T2 daily routine disruptions. Previous evidence has nevertheless suggested the health-promoting behavioral benefits of self-efficacy^8,10^ and meaning in life^13,14^. Taken together, this discrepancy might suggest that the regularity of daily routines should be assessed and intervened separably from individual activities amid large-scale disasters or in the post-disaster period. For example, public health intervention could sequentially enhance regularity of daily routines or restore disrupted routines, which form a solid behavioral foundation for facilitating positive cognitive adaptation such as self-efficacy and meaning-making and then health behaviors.

Several limitations warranted cautions. First, the mediators (i.e., self-efficacy, meaning-making, daily routine disruptions) and the outcome variables (i.e., probable depression and anxiety) in the path analyses were assessed cross-sectionally. Therefore, we cannot fully rule out possible bidirectional associations, although there were a wealth of theoretical and empirical evidence^7,14,15^ supporting a directional relationship between the mediators and the outcome variables. Relatedly, there could be unmeasured confounding variables that dilute an absolute causal conclusion. Second, the small effect sizes of certain significant paths and the wide confidence intervals of certain non-significant paths, although comparable with previous similar studies in COVID-19, could have limited the validity of the current findings. Third, probable psychiatric conditions were self-reported. Although we used validated measures that have been tested reliable across contexts and populations^38,40^, the current evidence could be cross-validated with clinical diagnoses or structured interviews. Fourth, cognitive adaptation could also be manifested in mental processes other than efficacious and meaningful thinking, such as self-esteem, perceived predictability, and optimism^5^, which await future investigations. Fifth, routine subgroups were summarized into single items, whereas different activities belonging to each subgroup could be specifically analyzed within future studies. Finally, the interval between the two assessments was short, i.e., six months, and our results should be interpreted within the timeframe of one-year post-COVID-outbreak. As COVID-19 transformed from an acute stressor to a chronic stressor^49^, the respective roles of cognitive and behavioral factors in adaptive functioning might also differ.

Notwithstanding the limitations, this study has largely benefited from the cross-lagged analyses based on a population-representative prospective dataset, which have provided methodological and statistical rigor to the inferences of directionality between cognitive and behavioral adaptation. Overall consistency in patterns of probable depression and anxiety further supported the reliability of our conclusions. On a broader sense, the current study contributed preliminary insights to the interrelating cognitive and behavioral processes in psychological adjustment. Specifically, adjustment could warrant better mental health outcomes by beginning with regularizing daily routine behaviors followed by sustaining or gaining cognitive adaptation such as efficacious and meaningful thinking in order to achieve resilience under chronic stress and trauma. Moving forward, an in-depth understanding of the cognitive and behavioral processes, as well as their interactions, should be achieved with the investigation of additional cognitive and behavioral elements other than those (self-efficacy, meaning-making, and routine disruptions) included in the current study, such as self-esteem^5^, health behaviors^50^, and the ability to concentrate on individual work^51^. Experimental studies and clinical trials will further inform the clinical knowledge base for the cognitive-behavioral stream in psychotherapies. A more comprehensive consideration of additional diagnostic outcomes is necessary to determine whether these processes are truly shared across psychiatric conditions.

### Supplementary information

Supplementary Material

Editorial Policy Checklist

Reporting Summary

## Data Availability

The datasets used and/or analyzed during the current study are available from the corresponding author upon request.

## Code availability

The codes for analyses are available from the corresponding author upon request. All codes are written within M*plus* (Version 8.3) for the present study.
