# Supplementary material for: Investigating the reciprocity between cognition and behavior in adaptation to large-scale disasters
Source: Npj Ment Health Res. 2023 Dec 4;2:21. doi: 10.1038/s44184-023-00037-8 (PMC10955933; doi:10.1038/s44184-023-00037-8)
Supplement: Supplementary file 1 — Supplementary Material [file 44184_2023_37_MOESM1_ESM.docx]

**SUPPLEMENTARY NOTE 1** Detailed sampling procedure between March and August 2021 (T1).

This current study was part of an epidemiological cohort study which started in February–July 2020. Upon obtaining the approval from the Ethics Committee of The Education University of Hong Kong, the Centre for Communication and Public Opinion Survey of The Chinese University of Hong Kong and Hong Kong Public Opinion Research institute were contracted to conduct telephone surveys. Random digit dialing was employed based on a dual-frame sampling approach with both landline and mobile phone numbers (50% each) drawn from the databases released by the Hong Kong Communication Authority. Interviews were conducted with eligible respondents, who were (1) Hong Kong Chinese, (2) 15 years of age or older, and (3) Cantonese-speaking from 2 pm to 10 pm on both weekdays and weekends. Verbal informed consent was obtained prior to each interview. If multiple eligible members were identified in a successfully contacted household through landline phone calls, the one with the closest birthday to the interview date was selected. Further attempts were made for numbers with responses as “no answer”, “busy”, or “eligible respondent not at home”.

During March and August 2021 (i.e., T1 of the current study), a total of 1964 respondents were invited. 1964 telephone numbers were attempted, 202 (10.3%) were ineligible for inclusion (i.e., invalid number or eligible respondents passed away) and the numbers of unknown eligible was 325 (16.5%). Among 1437 (73.2%) eligible successfully contacted numbers, 1318 (91.7%) surveys were successfully completed, and 119 (8.3%) refused.

**SUPPLEMENTARY NOTE 2** Detailed sampling procedure between September 2021 and February 2022 (T2).

During September 2021 and February 2022 (i.e., T2 of the current study), a total of 1333 respondents were invited. 1333 telephone numbers were attempted, 10 (0.75%) were ineligible for inclusion (i.e., invalid number) and the numbers of unknown eligible was 403 (30.23%). Among 920 (69.02%) eligible successfully contacted numbers, 906 (98.48%) surveys were successfully completed, and 14 (1.52%) refused.

| Response rate = | Completed / [Known eligibles + Unknown eligibles × Eligibles / (Eligibles + Ineligibles)] |
| --- | --- |
| Cooperation rate = | Completed / Known eligibles |

**SUPPLEMENTARY NOTE 3** Confirmatory factor analyses evaluating the structural validity of single composite measures for cognitive adaptation and routine disruptions.

Confirmatory factor analysis (CFA) was conducted to evaluate the structural validity of the single composite measure with lavaan package in R [1]. Model fit was evaluated using comparative fit index (CFI), Tucker Lewis index (TLI), root mean square error of approximation (RMSEA), and standardized root mean square residual (SRMR). CFI and TLI values ≥ 0.90 and RMSEA and SRMR values ≤ 0.08 indicate a good fit [2].

For cognitive adaptation, we tested a bifactor measurement model of the items of self-efficacy and meaning making in which the common variance of the items was partitioned into a general factor (cognitive adaptation) and group factors (nuisance dimensions due to the same origin scale). Results showed excellent model fit of the bifactor model (T1: CFI = 0.973, TLI = 0.960, RMSEA = 0.061 (90% CI 0.054, 0.069), and SRMR = 0.070; T2: CFI = 0.990, TLI = 0.986, RMSEA = 0.034 (90% CI 0.026, 0.043), and SRMR = 0.028) and in general strong factor loadings between the cognitive adaptation factor and each item at both T1 (range: 0.262–0.829, mean: 0.483, median: 0.469) and T2 (range: 0.268–0.806, mean: 0.481, median: 0.426), supporting the generation of a composite score to represent cognitive adaptation.

For routine disruptions, a single factor model was examined on the routine items to test the model fitness since that the items were from the same instrument. Results showed good model fit of the measurement model at both T1 (CFI = 0.970, TLI = 0.950, RMSEA = 0.063 (90% CI 0.049, 0.077), and SRMR = 0.035) and T2 (CFI = 0.985, TLI = 0.975, RMSEA = 0.045 (90% CI 0.030, 0.060), and SRMR = 0.025). Factor loadings between the single factor and items of routine disruptions were strong over time (T1: range: 0.344–0.735, mean: 0.556, median: 0.594; T2: range: 0.296–0.758, mean: 0.572, median: 0.601), lending support to the generation of a composite score to represent routine disruptions.

**References**

1. Rosseel, Y. Lavaan: An R package for structural equation modeling and more. *J. Stat. Softw.* **48**, 1–36 (2012).
2. Hu, L. T. & Bentler, P. M. Cutoff criteria for fit indexes in covariance structure analysis: conventional criteria versus new alternatives. *Struct. Equ. Modeling.* **6**, 1–55 (1999).

**SUPPLEMENTARY TABLE 1** Cross-lagged analyses examining the autoregressive and cross-lagged effects of cognitive adaptation, routine disruptions, and depressive symptoms.

| **Effect** | **Variables†** | ***β* [95% CI]** |
| --- | --- | --- |
| Autoregressive | T1 Cognitive adaptation → T2 Cognitive adaptation | 0.531 [0.465, 0.597]*** |
|  | T1 Routine disruptions → T2 Routine disruptions | 0.557 [0.491, 0.624]*** |
|  | T1 Depressive symptoms → T2 Depressive symptoms | 0.605 [0.556, 0.654]*** |
| Cross-lagged | T1 Cognitive adaptation → T2 Routine disruptions | –0.026 [–0.086, 0.034] |
|  | T1 Routine disruptions → T2 Cognitive adaptation | –0.075 [–0.142, –0.008]* |
|  | T1 Cognitive adaptation → T2 Depressive symptoms | –0.046 [–0.101, 0.009] |
|  | T1 Routine disruptions → T2 Depressive symptoms | 0.095 [0.032, 0.157]** |
|  | T1 Depressive symptoms → T2 Cognitive adaptation | –0.049 [–0.119, 0.021] |
|  | T1 Depressive symptoms → T2 Routine disruptions | 0.046 [–0.015, 0.107] |

Note. * *p* < 0.050, ** *p* < 0.010, *** *p* < 0.001. Model fit indices: RMSEA = 0.034, 90% CI [0.014, 0.052], SRMR = 0.024, CFI = 0.994, TLI = 0.975.

† Depressive symptoms, cognitive adaptation, and routine disruptions were continuous variables.

**SUPPLEMENTARY TABLE 2** Cross-lagged analyses examining the autoregressive and cross-lagged effects of cognitive adaptation, routine disruptions, and anxiety symptoms.

| **Effect** | **Variables†** | ***β* [95% CI]** |
| --- | --- | --- |
| Autoregressive | T1 Cognitive adaptation → T2 Cognitive adaptation | 0.553 [0.466, 0.600]*** |
|  | T1 Routine disruptions → T2 Routine disruptions | 0.566 [0.500, 0.632]*** |
|  | T1 Anxiety symptoms → T2 Anxiety symptoms | 0.574 [0.517, 0.630]*** |
| Cross-lagged | T1 Cognitive adaptation → T2 Routine disruptions | –0.031 [–0.092, 0.030] |
|  | T1 Routine disruptions → T2 Cognitive adaptation | –0.081 [–0.146, –0.016]* |
|  | T1 Cognitive adaptation → T2 Anxiety symptoms | –0.040 [–0.098, 0.017] |
|  | T1 Routine disruptions → T2 Anxiety symptoms | 0.093 [0.025, 0.161]** |
|  | T1 Anxiety symptoms → T2 Cognitive adaptation | –0.035 [–0.102, 0.031] |
|  | T1 Anxiety symptoms → T2 Routine disruptions | 0.021 [–0.039, 0.082] |

Note. * *p* < 0.050, ** *p* < 0.010, *** *p* < 0.001. Model fit indices: RMSEA = 0.034, 90% CI [0.014, 0.053], SRMR = 0.022, CFI = 0.994, TLI = 0.973.

† Anxiety symptoms, cognitive adaptation, and routine disruptions were continuous variables.

**SUPPLEMENTARY TABLE 3** Path analyses examining the mutual mediating mechanisms of cognitive adaptation and routine disruptions on depressive/anxiety symptoms.

| **Outcome**† | **Direct/Indirect effects**† | **Direct/Indirect effect *β* [95% CI]** |
| --- | --- | --- |
| T2 Depressive symptoms | T1 Cognitive adaptation | 0.045 [–0.018, 0.109] |
|  | T1 Cognitive adaptation → T2 Routine disruptions | –0.006 [–0.015, 0.004] |
|  | T1 Routine disruptions | –0.001 [–0.072, 0.070] |
|  | T1 Routine disruptions → T2 Cognitive adaptation | 0.017 [0.005, 0.029]** |
| T2 Anxiety symptoms | T1 Cognitive adaptation | 0.049 [–0.019, 0.117] |
|  | T1 Cognitive adaptation→ T2 Routine disruptions | –0.005 [–0.014, 0.004] |
|  | T1 Routine disruptions | 0.003 [–0.077, 0.083] |
|  | T1 Routine disruptions → T2 Cognitive adaptation | 0.011 [0.000, 0.021]^ |

Note. ** *p* < 0.010, ^ *p* = 0.058. Model fit indices (Depressive symptoms): RMSEA = 0.031, 90% CI [0.000, 0.058], SRMR = 0.020, CFI = 0.996, TLI = 0.987; Model fit indices (Anxiety symptoms): RMSEA = 0.039, 90% CI [0.009, 0.068], SRMR = 0.018, CFI = 0.994, TLI = 0.979.

† Depressive symptoms, anxiety symptoms, cognitive adaptation, and routine disruptions were continuous variables.

**SUPPLEMENTARY FIGURE 1** Path model for cognitive adaptation, routine disruptions, and psychopathology (Panel A: probable depression; Panel B: probable anxiety).

**
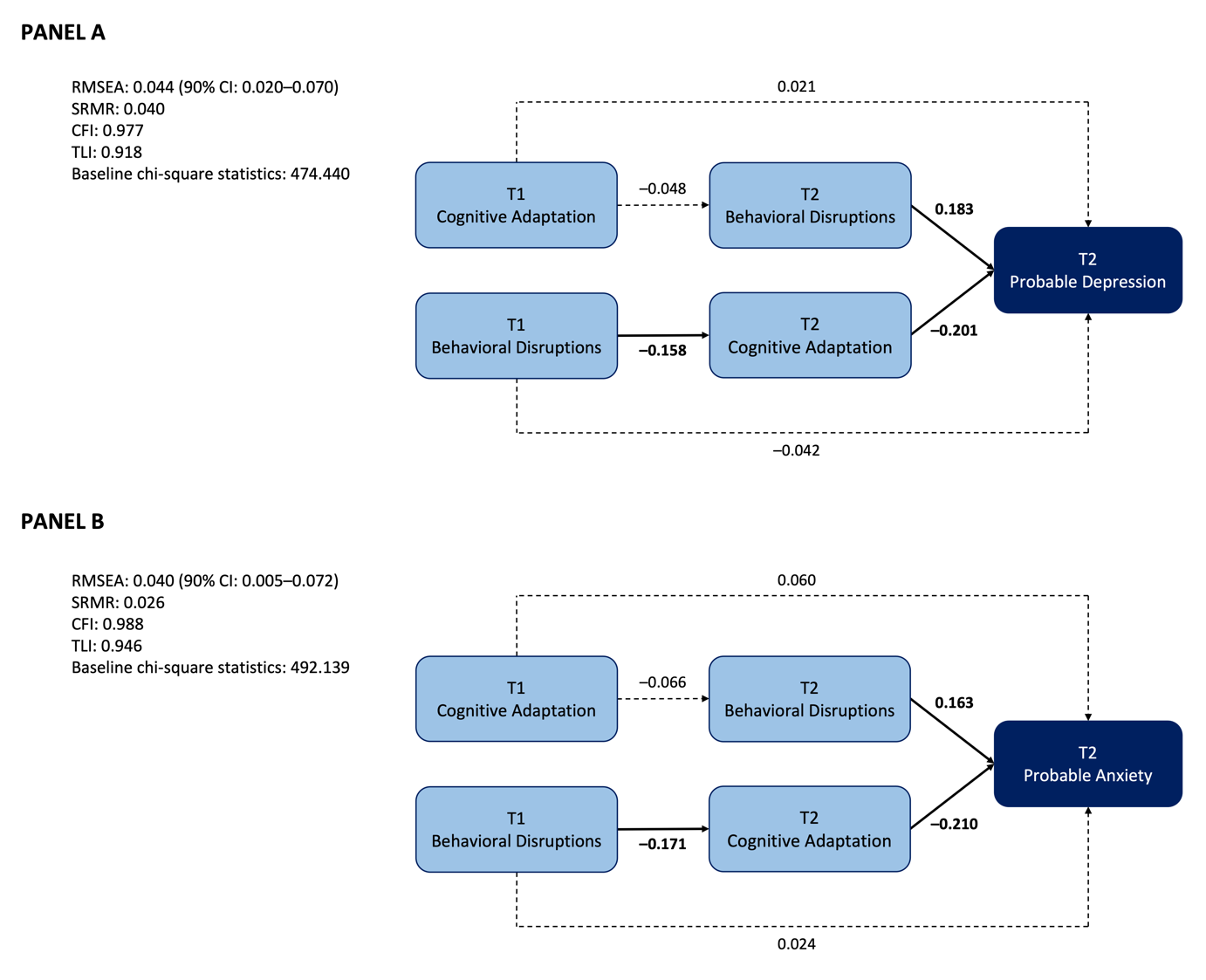
**

Note. Cognitive adaptation included self-efficacy and meaning making; routine disruptions included primary and secondary routine disruptions. Demographics covariates were adjusted for in the path model. The full figure including demographics covariates is available from the corresponding author.
