# Supplementary material for: Investigating the reciprocity between cognition and behavior in adaptation to large-scale disasters
Source: Npj Ment Health Res. 2023 Dec 4;2:21. doi: 10.1038/s44184-023-00037-8 (PMC10955933; doi:10.1038/s44184-023-00037-8)
Supplement: Supplementary file 3 — Reporting Summary [file 44184_2023_37_MOESM3_ESM.pdf]

nature portfolio

Corresponding author(s): Wai Kai HOU

Last updated by author(s): April 21st 2023

Reporting Summary

Nature Portfolio wishes to improve the reproducibility of the work that we publish. This form provides structure for consistency and transparency in reporting. For further information on Nature Portfolio policies, see our Editorial Policies and the Editorial Policy Checklist.

Statistics

For all statistical analyses, confirm that the following items are present in the figure legend, table legend, main text, or Methods section.

n/a	Confirmed	
☐	☒	The exact sample size (n) for each experimental group/condition, given as a discrete number and unit of measurement
☐	☒	A statement on whether measurements were taken from distinct samples or whether the same sample was measured repeatedly
☐	☒	The statistical test(s) used AND whether they are one- or two-sided<br>*Only common tests should be described solely by name; describe more complex techniques in the Methods section.*
☐	☒	A description of all covariates tested
☐	☒	A description of any assumptions or corrections, such as tests of normality and adjustment for multiple comparisons
☐	☒	A full description of the statistical parameters including central tendency (e.g. means) or other basic estimates (e.g. regression coefficient) AND variation (e.g. standard deviation) or associated estimates of uncertainty (e.g. confidence intervals)
☐	☒	For null hypothesis testing, the test statistic (e.g. F, t, r) with confidence intervals, effect sizes, degrees of freedom and P value noted<br>*Give P values as exact values whenever suitable.*
☐	☒	For Bayesian analysis, information on the choice of priors and Markov chain Monte Carlo settings
☐	☒	For hierarchical and complex designs, identification of the appropriate level for tests and full reporting of outcomes
☐	☒	Estimates of effect sizes (e.g. Cohen's d, Pearson's r), indicating how they were calculated

Our web collection on statistics for biologists contains articles on many of the points above.

Software and code

Policy information about availability of computer code

Data collection	No software was used.
Data analysis	MPlus version 8.3.

For manuscripts utilizing custom algorithms or software that are central to the research but not yet described in published literature, software must be made available to editors and reviewers. We strongly encourage code deposition in a community repository (e.g. GitHub). See the Nature Portfolio guidelines for submitting code & software for further information.

Data

Policy information about availability of data

All manuscripts must include a data availability statement. This statement should provide the following information, where applicable:

- Accession codes, unique identifiers, or web links for publicly available datasets
- A description of any restrictions on data availability
- For clinical datasets or third party data, please ensure that the statement adheres to our policy

The datasets used and/or analyzed during the current study available from the corresponding author on reasonable request.

Research involving human participants, their data, or biological material

Policy information about studies with human participants or human data. See also policy information about sex, gender (identity/presentation), and sexual orientation and race, ethnicity and racism.

Reporting on sex and gender	At T1, gender, age, marital status, education level, employment status, and monthly household income were recorded.
Reporting on race, ethnicity, or other socially relevant groupings	Telephone surveys were conducted among Hong Kong Chinese aged ≥15 years by interviewers who received formal training. The ethnicity was reported by the respondents.
Population characteristics	Demographics of the respondents (N=906) are summarized in Table 1.
Recruitment	Telephone surveys were conducted among Hong Kong Chinese aged ≥15 years by interviewers who received formal training. Oral informed consent was obtained from each participant prior to the survey. The sampling procedure of the current study closely followed that of other large-scale local prospective cohort studies.
Ethics oversight	The methods were performed in accordance with relevant guidelines and regulations and approved by The Education University of Hong Kong.

Note that full information on the approval of the study protocol must also be provided in the manuscript.

Field-specific reporting

Please select the one below that is the best fit for your research. If you are not sure, read the appropriate sections before making your selection.

☐ Life sciences ☒ Behavioural & social sciences ☐ Ecological, evolutionary & environmental sciences

For a reference copy of the document with all sections, see nature.com/documents/nr-reporting-summary-flat.pdf

Behavioural & social sciences study design

All studies must disclose on these points even when the disclosure is negative.

Study description	Quantitative, prospective design
Research sample	This was a prospective study with population-representative data collected at two timepoints, with a follow-up duration of six months for all respondents, March–August 2021 (T1), and September 2021–February 2022 (T2). The methods were performed in accordance with relevant guidelines and regulations and approved by The Education University of Hong Kong. Telephone surveys were conducted among Hong Kong Chinese aged ≥15 years by interviewers who received formal training. Oral informed consent was obtained from each participant prior to the survey. The sampling procedure of the current study closely followed that of other large-scale local prospective cohort studies42. The final sample included 906 individuals. The cooperation (eligible individuals invited) and response (invited individuals complying with acceptable standards) rates at T2 (98.48% and 68.64%) were good (Supplementary Materials 1–2).
Sampling strategy	This current study was part of an epidemiological cohort study which started in February–July 2020. Upon obtaining the approval from the Ethics Committee of The Education University of Hong Kong, the Centre for Communication and Public Opinion Survey of The Chinese University of Hong Kong and Hong Kong Public Opinion Research institute were contracted to conduct telephone surveys. Random digit dialing was employed based on a dual-frame sampling approach with both landline and mobile phone numbers (50% each) drawn from the databases released by the Hong Kong Communication Authority. Interviews were conducted with eligible respondents, who were (1) Hong Kong Chinese, (2) 15 years of age or older, and (3) Cantonese-speaking from 2 pm to 10 pm on both weekdays and weekends. Verbal informed consent was obtained prior to each interview. If multiple eligible members were identified in a successfully contacted household through landline phone calls, the one with the closest birthday to the interview date was selected. Further attempts were made for numbers with responses as "no answer", "busy", or "eligible respondent not at home."
Data collection	Telephone surveys were conducted among Hong Kong Chinese aged ≥15 years by interviewers who received formal training.
Timing	This was a prospective study with population-representative data collected at two timepoints, with a follow-up duration of six months for all respondents, March–August 2021 (T1), and September 2021–February 2022 (T2).
Data exclusions	No data were excluded.
Non-participation	During September 2021 and February 2022 (i.e., T2 of the current study), a total of 1333 respondents were invited. 1333 telephone numbers were attempted, 10 (0.75%) were ineligible for inclusion (i.e., invalid number) and the numbers of unknown eligible was 403 (30.23%). Among 920 (69.02%) eligible successfully contacted numbers, 906 (98.48%) surveys were successfully completed, and 14 (1.52%) refused.
Randomization	Participants were not allocated into experimental groups.

Reporting for specific materials, systems and methods

We require information from authors about some types of materials, experimental systems and methods used in many studies. Here, indicate whether each material, system or method listed is relevant to your study. If you are not sure if a list item applies to your research, read the appropriate section before selecting a response.

Materials & experimental systems

n/a	Involved in the study	
☒	☐	Antibodies
☒	☐	Eukaryotic cell lines
☒	☐	Palaeontology and archaeology
☒	☐	Animals and other organisms
☒	☐	Clinical data
☒	☐	Dual use research of concern
☒	☐	Plants

Methods

n/a	Involved in the study	
☒	☐	ChIP-seq
☒	☐	Flow cytometry
☒	☐	MRI-based neuroimaging
